# Two Japanese Cases of Breast Cancer That Developed Pembrolizumab-Induced Adrenal Insufficiency and Literature Review

**DOI:** 10.7759/cureus.78620

**Published:** 2025-02-06

**Authors:** Yuka Ozawa, Takaaki Tokito, Mariko Kikuchi, Hiroshi Katoh, Takafumi Sangai

**Affiliations:** 1 Breast and Thyroid Surgery, Kitasato University Hospital, Sagamihara, JPN

**Keywords:** adrenal insufficiency, early triple-negative breast cancer, immune-related adverse events, japanese, pembrolizumab

## Abstract

We report two cases of adrenal insufficiency (AI) occurring during neoadjuvant treatment with pembrolizumab for breast cancer. In the first case, a 53-year-old female presented with a chief complaint of poor oral intake and fatigue. In the second case, a 46-year-old female presented with a chief complaint of fever, poor oral intake, and general fatigue and was admitted with a diagnosis of pneumonia. Her symptoms did not improve during treatment for pneumonia. After that, two patients were diagnosed with pembrolizumab-induced adrenal insufficiency and were treated with hydrocortisone with improvement in their symptoms. AI due to pembrolizumab use is a relatively rare adverse event, but if it is detected late, it can be potentially life-threatening. In both cases, there were clear changes in the common terminology criteria for adverse events (CTCAE) grade at the time of diagnosis of AI. It may be useful for early detection of AI. The CTCAE version 5.0 was used to assess the severity of adverse events.

## Introduction

Recurrence and metastasis of triple-negative breast cancer (TNBC) are associated with poor prognosis, making the initial treatment aimed at controlling potential microscopic metastases through local and systemic therapies extremely important. The KEYNOTE-522 trial demonstrated that the addition of pembrolizumab significantly improved pathological complete response rates and event-free survival [1], leading to the use of pembrolizumab, an anti-PD-1 (Programmed cell Death-1) antibody, as neoadjuvant and adjuvant immunotherapy for high-risk early TNBC. Currently, pembrolizumab is used for many malignancies, such as lung cancer, urothelial carcinoma, and malignant melanoma, in addition to breast cancer. On the other hand, immune-related adverse events (irAEs) associated with pembrolizumab have been reported, and endocrine disorders, such as adrenal insufficiency (AI), which can be fatal, are one of the irAEs that should be monitored closely [2]. In the two cases we encountered, one presented with typical symptoms, allowing for early diagnosis and treatment intervention, while the other exhibited various symptoms, making diagnosis difficult. With this case report, we aim to contribute to the growing body of evidence and raise awareness among clinicians.

## Case presentation

Case 1

In the first case, a 53-year-old female with no significant past medical history noticed a lump in her right breast. She was diagnosed with right breast cancer (Invasive ductal carcinoma, Triple negative type, cT2N0M0 cStageIIA) and started neoadjuvant chemotherapy and immunotherapy. After the first cycle of pembrolizumab, carboplatin, and paclitaxel, the laboratory data showed hyperthyroidism and then hypothyroidism at the time of the third cycle. She was administered Levothyroxine. She also started complaining of nausea and mild shortness of breath, but both were considered Grade 1. Following her second cycle of pembrolizumab, epirubicin, and cyclophosphamide, she experienced worsening appetite loss and general fatigue (Grade 2). She was suspected of secondary AI and underwent a laboratory test (Table 1).

**Table 1:** Laboratory test results at the first visit and when diagnosed with AI AST: Aspartate aminotransferase; ALT: Alanine aminotransferase; CRP: C-reactive protein; TSH: Thyroid-stimulating hormone; FT3: Free triiodothyronine; FT4: Free thyroxine; ACTH: Adrenocorticotropic hormone

Laboratory Test	Result at the first visit	Result when diagnosed with AI	Reference Range
White Blood Cell	5.4 *10^3^/µL	4.5 *10^3^/µL	3.3-8.6 *10^3^/µL
Neutrophil	52.9%	45.3%	50-70 %
Eosinophil	3.0%	0.9%	2.0-5.0 %
Red Blood Cell	4.3 *10^6^/µL	3.3 *10^6^/µL	3.86-4.92 *10^6^/µL
Hemoglobin	12.9 g/dL	10.8 g/dL	11.6-14.8 g/dL
Platelet	34.6 *10^4^/µL	42.1 *10^4^/µL	15.8-34.8 *10^4^/µL
Protein	7.2 g/dL	6.5 g/dL	6.6-8.1 g/dL
AST	16 U/L	22 U/L	13-30 U/L
ALT	14 U/L	13 U/L	7-23 U/L
Creatinin	0.91 mg/dL	0.73 mg/dL	0.46-0.79 mg/dL
Sodium	144 mmol/L	140 mmol/L	138-145 mmol/L
Potassium	3.8 mmol/L	3.6 mmol/L	3.6-4.8 mmol/L
CRP	0.06 mg/dL	0.13 mg/dL	<0.14 mg/dL
Glucose	84 mg/dL	113 mg/dL	73-109 mg/dL
TSH	0.7 µIU/mL	14.1 µIU/mL	0.5-5.0 µIU/mL
FT3	2.78 pg/mL	2.54 pg/mL	2.3-4.0 pg/mL
FT4	1.35 ng/dL	0.88 ng/dL	0.9-1.7 ng/dL
ACTH	31.8 pg/mL	<2.0 pg/mL	7.2-63.3 pg/mL
Cortisol	17.8 µg/dL	0.32 µg/dL	7.07-19.6 µg/dL

As demonstrated in Table 1, the patient's adrenocorticotropic hormone (ACTH) and cortisol levels were significantly below the normal reference range, a key indicator of adrenal insufficiency. This finding, coupled with clinical symptoms, confirmed the diagnosis of pembrolizumab-induced AI. She was administered a physiological maintenance of hydrocortisone 15mg; her symptoms significantly improved the next day. After that, computerized tomography (CT) and magnetic resonance imaging (MRI) scans showed a clinical complete response (cCR), so pembrolizumab was not resumed. Instead, she underwent a right total mastectomy and sentinel lymph node biopsy. Postoperative pathology revealed no residue of malignancy. Currently, she is receiving pembrolizumab again while taking hydrocortisone.

Case 2

In the second case, a 46-year-old female with no significant past medical history was referred for further evaluation following a screening ultrasound. She was diagnosed with left breast cancer (Invasive ductal carcinoma, Triple negative type, cT2N0M0, cStage IIA) and started neoadjuvant chemotherapy and immunotherapy. After the introduction of pembrolizumab, carboplatin, and paclitaxel, she started complaining of nausea and fatigue, which were equivalent to Grade 1. After the first cycle of pembrolizumab, epirubicin, and cyclophosphamide, her symptoms worsened (Grade 2), and she developed a fever, so she was hospitalized (Table 2).

**Table 2:** Laboratory test results at the first visit and at the time of hospitalization AST: Aspartate aminotransferase; ALT: Alanine aminotransferase; CRP: C-reactive protein; TSH: Thyroid-stimulating hormone; FT3: Free triiodothyronine; FT4: Free thyroxine; ACTH: Adrenocorticotropic hormone *These two items were tested on the third day of hospitalization.

Laboratory Test	Result at the first visit	Result at the time of hospitalization	Reference Range
White Blood Cell	5.4 *10^3^/µL	13.7 *10^3^/µL	3.3-8.6 *10^3^/µL
Neutrophil	46%	44%	50-70 %
Eosinophil	18%	48%	2.0-5.0 %
Red Blood Cell	4.31 *10^6^/µL	4.45 *10^6^/µL	3.86-4.92 *10^6^/µL
Hemoglobin	11.7 g/dL	13.5 g/dL	11.6-14.8 g/dL
Platelet	32.6 *10^4^/µL	22.9 *10^4^/µL	15.8-34.8 *10^4^/µL
Protein	6.6 g/dL	6.1 g/dL	6.6-8.1 g/dL
AST	32 U/L	26 U/L	13-30 U/L
ALT	27 U/L	19 U/L	7-23 U/L
Creatinin	0.73 mg/dL	0.47 mg/dL	0.46-0.79 mg/dL
Sodium	140 mmol/L	126 mmol/L	138-145 mmol/L
Potassium	4.2 mmol/L	3.8 mmol/L	3.6-4.8 mmol/L
CRP	<0.03 mg/dL	2.44 mg/dL	<0.14 mg/dL
Glucose	137 mg/dL	113 mg/dL	73-109 mg/dL
TSH	2.09 µIU/mL	3.88 µIU/mL	0.5-5.0 µIU/mL
FT3	2.64 pg/mL	4.07 pg/mL	2.3-4.0 pg/mL
FT4	1.3 ng/dL	1.77 ng/dL	0.9-1.7 ng/dL
ACTH	41.8 pg/mL	<2.0 pg/mL(*)	7.2-63.3 pg/mL
Cortisol	8.51 µg/dL	<0.2 µg/dL(*)	7.07-19.6 µg/dL

**Figure 1:**
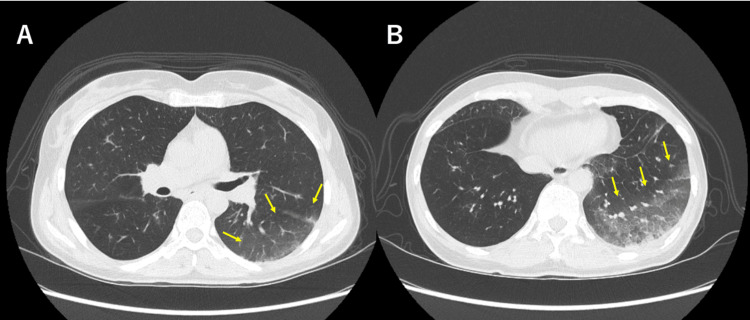
Chest CT scan

At the time of hospitalization, she had a productive cough and required low-dose oxygen. A chest CT scan revealed a ground-glass opacity in the dorsal segment of the left upper lobe (Figure 1A) and an infiltrative shadow in the dorsal segment of the left lower lobe (Figure 1B).

She was suspected of bronchopneumonia based on these findings. No clear distant metastasis was observed. She was diagnosed with aspiration pneumonia and received antibiotic treatment. On day three of hospitalization, she continued to experience poor appetite and general fatigue. In addition to the poor response to antibiotic therapy and persistent findings of hyponatremia and eosinophilia on blood tests raised, she was suspected of secondary AI and underwent additional laboratory tests (Table 2). As demonstrated in Table 2, the patient's ACTH and cortisol levels were significantly below the normal reference range. This finding and clinical symptoms confirmed the diagnosis of pembrolizumab-induced AI. A pituitary MRI did not reveal any abnormality or lesion. She was administered a stress-dose glucocorticoid replacement for three days. She gradually tapered glucocorticoid and transitioned to a physiological maintenance dose of hydrocortisone 15mg, immediately improving her appetite and energy level. She was discharged on day eleven of admission. After that, CT and MRI scans showed a cCR, so pembrolizumab was not resumed. Instead, she underwent a left total mastectomy and sentinel lymph node biopsy. Postoperative pathology revealed an invasive residual disease (ypT1aN0).

## Discussion

Currently, pembrolizumab is a therapeutic agent that binds to PD-1, which is involved in regulating autoimmune responses. This binding releases the brakes on the immune system by blocking inhibitory signals, reactivating T cells to increase their attack on tumors [3]. However, because it is a drug related to the body's autoimmune response, immune checkpoint inhibitors (ICIs) such as pembrolizumab can disrupt the normal regulation of the immune system, leading to the development of autoimmune diseases. These immune-related side effects are referred to as irAEs [4]. In fact, molecules involved in antigen presentation on T cells are expressed throughout the body so that irAEs can occur systemically. However, reports of these events are relatively more common in the skin, gastrointestinal tract, and endocrine system. There are several reports regarding the mechanisms of onset for some organs, such as the skin [5], thyroid [6], and pituitary gland [7-8]. However, there are no reports confirming the mechanisms of onset for adrenal cortical insufficiency.

Adrenal insufficiency as an irAE is observed in approximately 2.6% of patients receiving ICI treatment, making it a relatively rare adverse event [1]. Clinical symptoms of AI include fever, fatigue, and gastrointestinal symptoms. However, cases that reach grade 3 or higher are rare, occurring in approximately 1.0% of patients [1]. A search on the Japanese medical database Ichushi Web using the keywords "Pembrolizumab" and "adrenal cortical insufficiency" yielded 20 relevant articles. All of the articles were related to lung cancer, urological malignancies, gastrointestinal cancers, and malignant melanoma, and no case reports related to breast cancer were found. Out of the 20 articles, 12 case reports described the symptoms that led to the discovery of adrenal cortical insufficiency [9-16,18,19]. The most commonly reported symptoms were fatigue (75%) and poor appetite (50%). One of these reports indicated that the severity of the symptoms was equivalent to Grade 2 (Table 3).

**Table 3:** Symptoms from reported cases in the Japanese medical database CBDCA: Carboplatin; PEM: Pemetrexed; PTX: Paclitaxel; EC: Epirubicin and cyclophosphamide * : A case diagnosed with hyponatremia during chemotherapy for lung cancer, complicated by ACTH deficiency and adrenal insufficiency [Article in Japanese]. The 52nd Clinical Fluid Research Meeting); Nov 21, 2020.

Case No.	Age	Sex	Site of primary tumor	Stage IV Y/N	Drug types	Symptoms that led to the diagnosis	Reference
1	52	M	Lung	Y	CBDCA＋PEM＋Pembrolizmab	Fatigue	*
2	54	M	Lung	N	Pembrolizumab	Fatigue	[9]
3	77	M	Colon	Y	Pembrolizumab	Fatigue, poor appetite, and dry mouth	[10]
4	87	F	Colon	Y	Pembrolizumab	Poor appetite, shortness of breath, and depression	[11]
5	75	M	Skin	Y	Pembrolizumab	Fatigue and poor appetite	[12]
6	70	M	Lung	Y	Pembrolizumab	Fatigue and fever	[13]
7	59	M	Lung	Y	Pembrolizumab	Fatigue (Grade 2)	[14]
8	72	M	Lung	N	Pembrolizumab	Fatigue, poor appetite and diarrhea	[15]
9	74	M	Renal pelvis	N	Pembrolizumab	Fatigue and poor appetite	[16]
10	78	M	Renal pelvis	N	Pembrolizumab	Fatigue	[16]
11	70	M	Ureter	Y	Pembrolizumab	Fatigue and poor appetite	[18]
12	79	M	Lung	Y	Pembrolizumab	Consciousness disturbance	[19]
13	53	F	Breast	N	Pembrolizumab＋CBDCA＋PTX→Pembrolizumab＋EC	Fatigue and poor appetite (Grade 2)	Our case 1
14	46	F	Breast	N	Pembrolizumab＋CBDCA＋PTX→Pembrolizumab＋EC	Fatigue and poor appetite (Grade 2)	Our case 2

Additionally, cases with hyponatremia and eosinophilia were notable in the blood biochemical findings. Specifically, hyponatremia was observed in 10 out of 14 cases (71%; Grade 1/2/3/4, 3/0/6/1 cases, respectively). In fact, there are several studies suggesting that eosinophilia and hyponatremia are likely to be biomarkers indicative of AI [17].

In the two cases discussed, the CTCAE grade reached Grade 2 at the time of diagnosis of AI. There was a clear change in the grading of the symptoms following treatment. In the second case, pneumonia complicates the condition, which masks the typical symptoms of AI, leading to a delay in treatment intervention. If AI had been suspected at the stage when there was a change in the Grade and adrenal function tests had been added to the blood test, it is possible that treatment intervention could have been initiated at an earlier stage. Moreover, the blood tests at the time of hospitalization already showed eosinophilia and hyponatremia, and the biochemical findings suggested the possibility of AI.

In patients undergoing chemotherapy, side effects such as poor appetite and fatigue are commonly observed, which makes it possible to overlook AI caused by irAEs in clinical practice. As a trigger for suspecting AI, it is essential to carefully monitor changes in the severity of symptoms, reference the CTCAE grade, and conduct a more thorough patient interview to ensure timely recognition and appropriate management. Additionally, biochemical findings such as blood cell counts and electrolyte abnormalities can also help identify AI. Therefore, it is important to check these alongside the adverse symptoms to aid in early detection.

## Conclusions

We presented two cases of pembrolizumab-induced adrenal insufficiency. AI can often be masked by the side effects of chemotherapy, making it important to monitor changes in CTCAE grade and biochemical findings closely. It is particularly crucial to suspect AI in cases of grade 2 or higher fatigue, poor oral intake, or hyponatremia.
